# The main pulmonary artery in adults: a controlled multicenter study with assessment of echocardiographic reference values, and the frequency of dilatation and aneurysm in Marfan syndrome

**DOI:** 10.1186/s13023-014-0203-8

**Published:** 2014-12-10

**Authors:** Sara Sheikhzadeh, Julie De Backer, Neda Rahimian Gorgan, Meike Rybczynski, Mathias Hillebrand, Helke Schüler, Alexander M Bernhardt, Dietmar Koschyk, Peter Bannas, Britta Keyser, Kai Mortensen, Robert M Radke, Thomas S Mir, Tilo Kölbel, Peter N Robinson, Jörg Schmidtke, Jürgen Berger, Stefan Blankenberg, Yskert von Kodolitsch

**Affiliations:** Centre of Cardiology and Cardiovascular Surgery, University Medical Center Hamburg-Eppendorf, Martinistraße 52, 20246 Hamburg, Germany; Department of Diagnostic and Interventional Radiology, University Hospital Eppendorf, Hamburg, Germany; Department of Medical Biometry and Epidemiology, University Hospital Eppendorf, Hamburg, Germany; Centre for Medical Genetics, University Hospital Ghent, Ghent, Belgium; Institute of Human Genetics, Hannover Medical School, Hannover, Germany; Medizinische Klinik II / Kardiologie, Angiologie, am Universitätsklinikum Schleswig-Holstein, Campus Lübeck, Germany; Department für Kardiologie und Angiologie, Universitätsklinikum Münster, Münster, Germany; Institute of Medical Genetics, Charité Universitätsmedizin Berlin, Berlin, Germany

**Keywords:** Pulmonary artery, Marfan syndrome, *FBN1*, Echocardiography, Reference values

## Abstract

**Background:**

Echocardiographic upper normal limits of both main pulmonary artery (MPA) diameters (MPA-d) and ratio of MPA to aortic root diameter (MPA-r) are not defined in healthy adults. Accordingly, frequency of MPA dilatation based on echocardiography remains to be assessed in adults with Marfan syndrome (MFS).

**Methods:**

We enrolled 123 normal adults (72 men, 52 women aged 42 ± 14 years) and 98 patients with MFS (42 men, 56 women aged 39 ± 14 years) in a retrospective cross-sectional observational controlled study in four tertiary care centers. We defined outcome measures including upper normal limits of MPA-d and MPA-r as 95 quantile of normal persons, MPA dilatation as diameters > upper normal limits, MPA aneurysm as diameters >4 cm, and indication for surgery as MPA diameters >6 cm.

**Results:**

MPA diameters revealed normal distribution without correlation to age, sex, body weight, body height, body mass index and body surface area. The upper normal limit was 2.6 cm (95% confidence interval (CI) =2.44-2.76 cm) for MPA-d, and 1.05 (95% CI = .86–1.24) for MPA-r. MPA dilatation presented in 6 normal persons (4.9%) and in 68 MFS patients (69.4%; *P* < .001), MPA aneurysm presented only in MFS (15 patients; 15.3%; *P* < .001), and no patient required surgery. Mean MPA-r were increased in MFS (*P* < .001), but ratios >1.05 were equally frequent in 7 normal persons (5%) and in 8 MFS patients (10.5%; *P* = .161). MPA-r related to aortic root diameters (*P* = .042), reduced left ventricular ejection fraction (*P* = .006), and increased pulmonary artery systolic pressures (*P* = .040). No clinical manifestations of MFS and no *FBN1* mutation characteristics related to MPA diameters.

**Conclusions:**

We established 2.6 cm for MPA-d and 1.05 for MPA-r as upper normal limits. MFS exhibits a high prevalence of MPA dilatation and aneurysm. However, patients may require MPA surgery only in scarce circumstances, most likely because formation of marked MPA aneurysm may require LV dysfunction and increased PASP.

## Background

The old Ghent nosology considered dilatation of the main pulmonary artery (MPA) as a diagnostic criterion of Marfan syndrome (MFS) [1] but data on the prevalence of MPA dilatation or aneurysm remained both scarce and conflicting in MFS [2-5]. Most likely, little attention has been paid to MPA because cardiovascular complications of MFS usually result only from rupture or dissection of the aortic root [6] or from ventricular arrhythmia [7,8]. Moreover, cardiovascular criteria of MFS are usually assessed with transthoracic echocardiography, but in adults upper normal limits of diameters of the MPA (MPA-d) remain to be defined on echocardiography [9]. As a consequence, there is no current data on the echocardiographic prevalence of MPA dilatation in MFS patients, and for this reason the revised Ghent nosology removed MPA dilatation from the list of diagnostic criteria [9]. Some case reports highlight that MPA dilatation may lead to severe complications in MFS [10], and current magnetic resonance imaging (MRI) and computed tomography (CT) studies suggest that pathology of the MPA is frequent in MFS [3-5,11,12]. Hence, echocardiographic examination of the MPA may be important in the evaluation of adults with MFS to further elucidate the diagnostic and prognostic implications of MPA dilatation.

Dilatation of the MPA is present with MPA-d above upper normal limits, where CT and MRI studies identified diameters between 2.8 cm and 3.2 cm as upper limits [4,13-15]. Moreover, the MPA ratio (MPA-r) is defined as a ratio of MPA-d to aortic root diameter [14]. Usually, the diameter of MPA equals the size of the proximal aorta, and hence CT or MRI studies of pulmonary hypertension (PH) identify ratios >1 as a marker of MPA dilatation [14,16-19]. The largest study of reference values of MPA is the Framingham study which used non-contrast cardiac CT in 706 normal adults. This study established 2.9 cm in men and 2.7 cm in women as sex-specific normative reference values for MPA-d and 0.9 for MPA-r [14]. Aneurysms of MPA are defined with MPA-d >4 cm [20,21]. Such aneurysms are reported in 1 of 14,000 autopsies [22]. Criteria for surgery of MPA aneurysms are not firmly established, but surgeons agree to operate upon in most patients with symptomatic MPA aneurysms or in aneurysms with MPA-d >6 cm [20,21,23].

Normative MPA-d and MRA-r values on CT and MRI do not apply to transthoracic echocardiography for various reasons. First, echocardiographic MPA-d and aortic root diameters are measured at other sites of the vessel than on CT or MRI, which may infer substantial differences of measurements because of the asymmetric anatomy of both MPA and the aortic root [2,5,24]. Second, measurements on CT and MRI are usually obtained as outer diameters of the vessel rather than with the leading-edge method [24-26]. Third, compared to CT and MRI, transthoracic echocardiography has inherent limits such as measurement of MPA-d only in parallel and not perpendicular to the direction of the scan plane [27], inadequate images due to chest wall deformities in MFS patients which is reported in 5 to 23 percent [28], and the difficulty to obtain adequate imaging of the pulmonary root.

MPA aneurysms usually occur in the setting of PH, and they have been reported in PH group 1 [29] including PH related to congenital heart disease [30], where patent ductus arteriosus is the most frequently reported underlying pathology of the MPA aneurysms [31,32], PH group 2, including acquired heart valve disease [20], and PH group 3 including chronic obstructive pulmonary disease (COPD) [33]. Some MPA aneurysms arise in the absence of PH, and they may have a more benign prognosis than high pressure MPA aneurysms [30,32]. These low pressure MPA aneurysms may arise from pulmonary valve stenosis [20,34], vasculitis such as Behcet’s disease [35] or giant cell arteritis [36], infections such as tuberculosis [37], syphilis [38,39] and septic emboli [22], extravascular [40] or endovascular trauma [31,41], or degenerative diseases including atherosclerosis [42] and media degeneration [43]. Some “idiopathic” MPA aneurysms may form without any of these causes [20,23,44].

MPA aneurysms are usually asymptomatic but they may lead to symptoms from right heart failure secondary to pulmonary regurgitation or from compression of the trachea or bronchi, or from pulmonary emboli, which may form in the enlarged MPA [20,31]. MPA dissection is a rare complication of marked MPA aneurysm [21], which is often fatal because it leads to rupture rather than to formation of a re-entry [45].

MPA aneurysms may develop in patients with heritable connective tissue diseases such as Loeys-Dietz syndrome [46,47], and arterial tortuosity syndrome [48], but it is most typically observed in MFS [2-5,49-54]. MFS is an autosomal dominantly inherited disease with a phenotype that involves multiple organ systems and is caused by mutations in the gene coding for fibrillin-1, *FBN1*. MPA dilatation and aneurysm develop in the absence of common risk factors such as PH, or pulmonary valve or artery stenosis [1]. Instead, it is likely that MPA dilatation and aneurysm develop as a consequence of the weakness of the connective tissue with degeneration of the media layer of the pulmonary vessel similar to the process in the aortic root. As had been pointed out previously, the common embryonic origin of MPA and the aortic root may in part explain the common involvement of both vessels in pathologic processes [3,55], where this common involvement is also the reason why MFS is considered an absolute contraindication for usage of the pulmonary root as an autograft in a Ross operation [56,57].

We performed this retrospective cross-sectional observational controlled study with the goal to identify upper normal limits of MPA-d on echocardiography. Moreover, we intended to use these upper normal limits to assess frequency of MPA dilatation, MPA aneurysm, and the need for MPA surgery in adult MFS patients. Finally, we aimed to identify risk factors of MPA dilatation in MFS including specific clinical features of MFS and *FBN1* mutation characteristics.

## Methods

### Patients

The study was approved by the Hamburg and the Ghent ethics committee and it was carried out in compliance with the Helsinki Declaration, where patients gave consent to the inclusion into retrospective pseudonymized data analysis. First, we recruited normal individuals aged ≥16 years, with normal results on echocardiography including normal pulmonary artery systolic pressure (PASP) as estimated <30 mmHg from the tricuspid jet velocity obtained during echocardiographic evaluation [58], to establish normal echocardiographic MPA-d. These individuals underwent cardiovascular evaluation as check-up examinations, as examinations prior to non-cardiovascular operations, or for documentation of cardiovascular health prior to the initiation of medications with potential side-effects on the cardiovascular system. We excluded persons with a history or current symptoms of cardiovascular disease, lung disease such as COPD, pulmonary embolism, or cardiovascular risk factors comprising diabetes mellitus, chronic arterial hypertension, or with a long-standing history of smoking or current smoking. In this way we identified 73 persons from the University Hamburg-Eppendorf, 18 persons from the Ghent University, 11 persons from the University of Münster, and 21 persons from the University of Lübeck. Thus, echocardiographic recordings of a total of 123 normal persons comprising 72 men and 52 women at a mean age of 42 ± 14 years (range 16–77 years) were available for uniform assessment of normal MPA-d and MPA-r.

Second, we identified a total of 167 adult inhabitants of the Hamburg metropolitan area [59] with a causative *FBN1* gene mutation, who fulfilled diagnostic criteria of MFS according to the current Ghent nosology [9]. Of these 167 patients, we excluded 86 because original echocardiographic recordings were not available, and 5 because we did not perform standardized MPA measurements. Of these remaining 76 patients we excluded 4 other patients, because MPA measurements were technically inadequate (5.2%). In addition, 26 patients were recruited in Ghent, who were recruited according to the same criteria as in Hamburg [9]. Thus, we identified a total of 98 patients with MFS for this study including 42 men and 56 women at a mean age of 39 ± 14 years (range 14–79 years).

### Clinical variables

**Figure 1:**
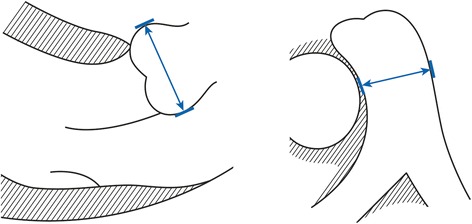
**We used 2-dimensional echocardiography to measure both, aortic root diameters and main pulmonary artery (MPA) diameters.** We performed all measurements at end-diastole perpendicular to the long axis of the vessel using the leading-edge method. We assessed aortic root diameters in the parasternal long-axis view at the level of the aortic sinuses (left panel) [62], and we measured MPA diameters in the parasternal short-axis view at the level of the maximum diameter above the root of the pulmonary artery (right panel).

We obtained age, body weight, body height, at the time of echocardiography, and we calculated body mass index (BMI) and body surface area (BSA) with the formula of Du Bois [60]. We re-evaluated standard 2-dimensional transthoracic echocardiographic recordings to assess left ventricular (LV) ejection fractions (LVEF) using Simpson’s rule [61], and 2-D-targeted M-mode for LV end-systolic (LVESD) and LV end-diastolic diameters (LVEDD) with normalization to BSA, all assessed according to current guidelines [61]. We measured aortic root diameters in the parasternal long-axis view at the level of the aortic sinuses at end-diastole using the leading-edge method as suggested recently (Figure 1, left panel) [62,63] with calculation of Z-scores according to the revised formula of Devereux et al. [64] where we calculated BSA according to Du Bois [60] (Tables 1 and 2).

**Table 1:** **Baseline characteristics**

**Variable**	**Normal persons**	**Marfan patients**	***P*** ^**1**^
	**(N = 123)**	**(N = 98)**	
Male gender	72 (59%)	42 (43%)	.022
Age (years)	42 ± 14 (16–77 )	39 ± 14 (14–79)	.113
Body weight (kg)	75 ± 14 (50–128)	74 ± 14 (45–115)	.398
Body height (m)	1.76 ± .1 (1.51–1.99)	1.85 ± .1 (1.58–2.10)	<.001
Body mass index (kg/m^2^)	24.2 ± 3.5 (16.3–39.9)	21.5 ± 3.6 (15.2–30.8)	<.001
Body surface area (DuBois; m^2^)	1.9 ± .21 (1.51–2.43)	1.96 ± .21 (1.51–2.51)	.040
Aortic root diameter (cm)^2^	3 ± .5 (1.9–4.2)	4.0 ± .7 (2–5.5)	<.001
Z-score of aortic root diameter	-.8 ± 1.6 (−6.2–1.9)	2.9 ± 2.3 (−4.4–7.4)	<.001
LV ejection fraction (%)	64 ± 6 (55–76)	55 ± 12 (21–78)	<.001
LVESD (mm/m^2^)	16 ± 3 (9–24)	18 ± 4 (10–36)	.005
LVEDD (mm/m^2^)	26 ± 3 (18–32)	27 ± 5 (20–48)	.009
Indexed left atrial diameter (mm/m^2^)	19 ± 3 (13–27)	19 ± 5 (8–32)	.339

LV identifies left ventricle; LVEDD, indexed left ventricular end-diastolic diameter; LVESD, indexed left ventricular end-systolic diameter, and MPA ratio, ratio of main pulmonary artery diameter/aortic root diameter. ^1^Fisher’s exact test for nominal and categorical data and the Mann–Whitney test for continuous data. ^2^We obtained aortic root diameters in 103 normal persons and in 76 patients with Marfan syndrome.

**Table 2:** **Correlation** ***r*** **of variables with the diameter of the main pulmonary artery (cm)**

	**Product–moment correlation coefficient** ***r*** **(** ***P*** **-value)** ^**1**^					
	**Normal persons**			**Marfan patients**		
**Variable**	**All** ^**2**^	**Male**	**Female**	**All**	**Male**	**Female**
	**(N = 123)**	**(N = 72)**	**(N = 51)**	**(N = 98)**	**(N = 42)**	**(N = 56)**
Age (years)	-.095 (.296)	-.01 (.929)	-.2 (.159)	.203 (.045)	.279 (.074)	.152 (.262)
Body weight (kg)	.172 (.057)	.013 (.913)	.293 (.037)	.115 (.261)	.125 (.429)	.133 (.327)
Body height (m)	.09 (.319)	.049 (.681)	.145 (.311)	.093 (.362)	.108 (.498)	.130 (.340)
Body mass index (kg/m^2^)	.141 (.121)	-.036 (.766)	.269 (.056)	.076 (.459)	.096 (.543)	.062 (.648)
Body surface area (DuBois; m^2^)	.149 (.099)	.032 (.789)	.262 (.063)	.120 (.240)	.142 (.369)	.146 (.282)

^1^ *P*-value for rho. ^2^The mean MPA diameters were 2.07 cm in normal males versus 2.05 cm in normal females (*P* = .747).

### MPA measurements and definitions

We measured MPA-d in the parasternal short-axis view at the level of the maximum diameter above the level of the pulmonary root at end-diastole [55] using the leading-edge method (Figure 1, right panel) [65], and we calculated the MPA-r as the ratio of the MPA-d and the aortic root diameter as measured at the level of the aortic sinuses (Figure 1, left panel) [14]. We defined upper normal limits of MPA-d and MPA-r as the 95 quantile of measurements of normal persons. Based on MPA measurements, we defined MPA dilatation as diameters > upper normal limits, MPA aneurysm as diameters >4 cm [20,21], and indication for surgery as MPA-d >6 cm (Table 3) [20,21].

**Table 3:** **Two-dimensional echocardiographic findings of the main pulmonary artery (MPA)**

**Variable**	**Normal persons**	**Marfan patients**	***P*** ^**1**^
	**(N = 123)**	**(N = 98)**	
MPA diameter (cm)	2.1 ± .3 (1.1–3.1)	3.1 ± .7 (1.9–4.7)	<.001
MPA dilatation (diameter > 2.6 cm)	6 (4.9%)	68 (69.4%)	<.001
MPA aneurysm (diameter > 4.0 cm)	0	15 (15.3%)	<.001
MPA diameter > 6.0 cm	0	0	
MPA ratio^2^	.7 ± .19 (.3–1.2)	.8 ± .21 (.5–1.5)	.001
MPA ratio > 1.05^2^	6 (5.8%)	8 (10.5%)	.161

LV identifies left ventricular; LVEDD, indexed left ventricular end-diastolic diameter; LVESD, indexed left ventricular end-systolic diameter, and MPA ratio, ratio of main pulmonary artery diameter/aortic root diameter. ^1^Fisher’s exact test for nominal and categorical data and the Mann–Whitney test for continuous data. ^2^We obtained aortic root diameters in 103 normal persons and in 76 patients with Marfan syndrome.

### MFS-related variables

We considered a family history of MFS in patients with a parent, child or sib who fulfilled diagnostic criteria of MFS. We established ectopia lentis with any unilateral or bilateral displacement of the lenses as documented on slit-lamp examination under full pupillary dilatation [9], or after operation for this entity. We documented pneumothorax with spontaneous pneumothorax on medical records [59], dural ectasia with lumbosacral dural ectasia according to the criteria of Habermann [66,67], striae distensae with striae that were not associated with pronounced weight changes or pregnancy, or with uncommon location such as the mid back, lumbar region, the upper arm, axillary region or thigh as documented during physical examination [9]. We diagnosed myopia with > −3 diopters on refractometry during ophthalmic examination or with > −3 diopters documented on the prescription of spectacles, mitral valve prolapse with posterior or anterior late systolic prolapse on M-mode or on 2-dimensional echocardiography from parasternal long axis views and apical 4-chamber views as leaflet displacement >2 mm [68]. We documented a systemic score ≥7 points as defined by revised Ghent criteria [9], where we displayed the score separately for skeletal features comprising the wrist sign, the thumb sign, pectus carinatum, pectus excavatum, chest asymmetry, hindfoot deformity, plain pes planus, protrusio acetabuli, reduced upper segment/lower segment ratio, increased arm/height ratio, scoliosis or thoracolumbar kyphosis, reduced elbow extension, and facial features as the skeletal score, and pneumothorax, skin striae, myopia > −3 diopters, and mitral valve prolapse as the non-skeletal score (Table 4) [69].

**Table 4:** **Relationship of variables with MPA diameter in 72 Marfan patients** ^**1**^

**Clinical variables**	**Regression coefficient (b)**	**95% confidence interval**	***P*** ^**2**^
Aortic root dilatation	-.118	-.491–.225	.531
Previous aortic root surgery	-.054	-.449–.342	.787
Aortic root diameter (cm)	.308	.011–.604	.042
Z-score of aortic root diameter	.035	-.051–.122	.414
LV ejection fraction (%)	-.021	-.036–.006	.006
LVESD (mm/m^2^)	.024	. -.018–.065	.258
LVEDD (mm/m^2^)	.043	-.007–.079	.021
PASP (mmHg)^2^	.023	.001–.045	.040
Family history of Marfan	.132	-.217–.482	.452
Ectopia lentis	-.183	-.531–.165	.298
Pneumothorax	-.133	-.617–.351	.585
Dural ectasia (Habermann)	.095	-.254–.443	.590
Striae distensae	-.021	-.331–.373	.906
Myopia > −3 diopters	-.294	-.691–.102	.143
Mitral valve prolapse	.224	-.427–-.020	.081
Systemic score ≥ 7 points	-.026	-.415–.364	.895
- Total systemic score points	-.010	-.076–.054	.764
- Skeletal score points	-.030	-.113–.053	.470
- Non-skeletal score points	.033	-.095–.161	.610
*FBN1* mutation characteristics			
PTC mutations	-.150	-.550–.249	.455
Splicing mutations	.208	-.422–.837	.513
Any mutation affecting Cys	.027	-.359–.412	.890
Missense affecting Cys	-.026	-.536–.484	.919
Any mutation in cbEGF	-.133	-.365–.262	.589
Missense in cbEGF	-.021	-.564–.606	.943
Any mutation in LTBP-bd	-.540	−1.240–.160	.128
Missense mutation in LTBP-bd	.018	−1.106–1.142	.974
Any mutation in exons 24–32	-.135	-.643–.373	.597
Missense in exons 24–32	-.014	-.654–.629	.965

cbEGF indetifies calcium-binding epidermal growth factor-like domain; coefficient, regression coefficient; Cys, cysteine; LV, left ventricular; LVEDD, indexed left ventricular end-diastolic diameter, LVESD, indexed left ventricular end-systolic diameter, LTBP-bd, latent TGF-beta-binding domain; missense, missense mutation; N, number; PASP, pulmonary artery systolic pressure; and PTC, premature termination codon. ^1^We analyzed on those 72 patients, whom we recruited in Hamburg. ^2^Linear regression analysis. ^2^PASP measurements were 22 ± 12 mmHg (range 6 – 49 mmHg).

### Molecular analysis

We amplified all 65 coding exons and intronic flanking splice-sites of *FBN1* (NM_000138.4) with polymerase chain reaction (PCR) from genomic deoxyribonucleic acid with previously published primers [70]. Subsequently we purified PCR products and sequenced with a Genetic Analyser (ABI 3130XL, Applied Biosystems Inc., Foster City, CA, USA). We detected gross deletions/duplications in the *FBN1* gene with multiplex ligation-dependent probe amplification (MLPA; SALSA® MLPA® kit, probemix P065 and P066, MRC Holland, Amsterdam, Netherlands). All *FBN1* gene nucleotide changes fulfilled ≥1 Ghent criteria of causality as defined by the current Ghent nosology [9].

### *FBN1* mutation characteristics

To assess *FBN1* mutation characteristics we compared both premature truncation codon-mutations, and splicing mutations versus all other mutations. In mutations with elimination or creation of a cysteine, with location in a calcium binding calcium binding epidermal growth factor-like (cbEGF) domain, or in a latent transforming-growth-factor beta–binding protein-like (LTBP) domain, or in exons 24–32, we first compared all mutations with the respective characteristic versus all other exon mutations, and second only missense mutations with the respective characteristic versus all other missense mutations (Table 4) [69,71].

### Data analysis

**Figure 2:**
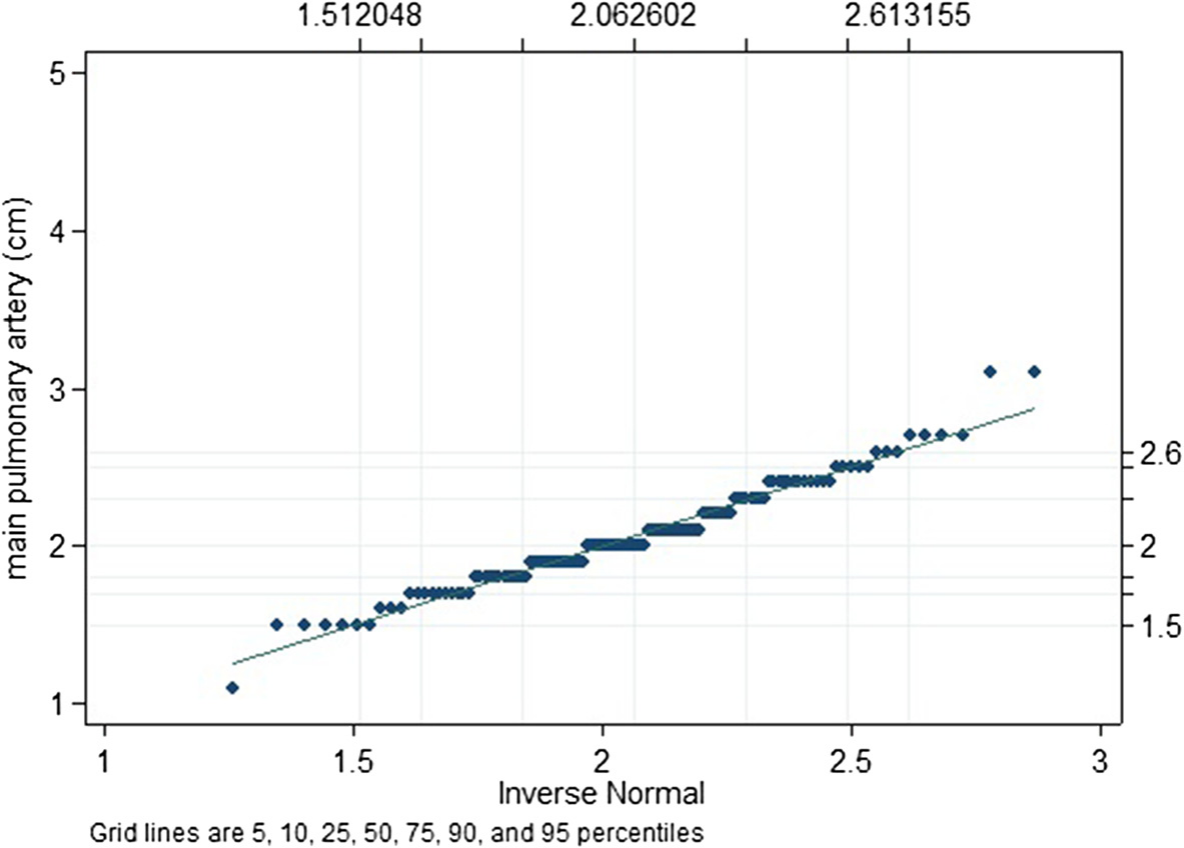
**The qnorm (Q-Q plot) plots the quantiles of MPA diameters (cm) of 123 normal adults against the quantiles of a normal distribution where the pattern of points in the plot is used to compare these two distributions.** The plot identifies normal distribution with only a slight deviation from normality at the upper tail. The Shapiro-Wilk W test for normal data corroborates normal distribution of MPA diameters yielding W = 0.98330, V = 1.641, z = 1.111, and *P* = .133.

We expressed quantitative data as means ± standard deviation (range), and qualitative data as numbers (percentage). We compared qualitative data with the Fisher’s exact test and quantitative data with the Mann–Whitney test (Tables 1 and 3). We employed the Shapiro-Wilk W test for normal data and the qnorm plot (Q-Q plot; Figure 2) to test for normal distribution of MPA-d [72], and we assessed the correlation of variables with MPA-d by means of the product–moment correlation coefficient *r* (Table 2). We used the quantile regression to estimate the 95-quantile and its 95%-confidence interval. We used the bootstrap technique to get robust estimation of the standard errors. Finally, we examined the relationship of clinical and molecular variables with MPA-d as dependent variable with linear regression analysis (Table 4). We considered *P*-values < .05 as significant. We used SPSS software (SPSS for Windows, Release 17.0, SPSS Inc. 1993 to 2007, Chicago, Illinois) and Stata 13 (Stata Statistical Software, Release 13; College Station, TX: StataCorp LP) for statistical analyses.

## Results

Baseline characteristics revealed less females (*P* = .022), smaller body-height, BSA (*P* = .040), aortic root diameters, aortic Z-scores (all *P* < .001), indexed LVESD (*P* = .005) and indexed LVEDD (*P* = .009) and higher BMI and LVEF (both *P* < .001) in normal adults than in MFS (Table 1).

MPA-d revealed normal distribution (Figure 2; *P* = .133) without correlation to age, body weight, body height, BMI and BSA both in normal adults and in MFS and with equal distribution of MPA diameters in men and women (*P* = .747; Table 2). The mean MPA-d in normal adults was 2.1 cm (95% confidence interval (CI) = 2.00–2.12 cm), and the upper normal limit of MPA-d was 2.6 cm (95% CI = 2.44-2.76 cm). The mean MPA-r in normal adults was .70 (95% CI = .67 – .74), and the upper limit of normal MPA-r was 1.05 (95% CI = .86– 1.24).

**Figure 3:**
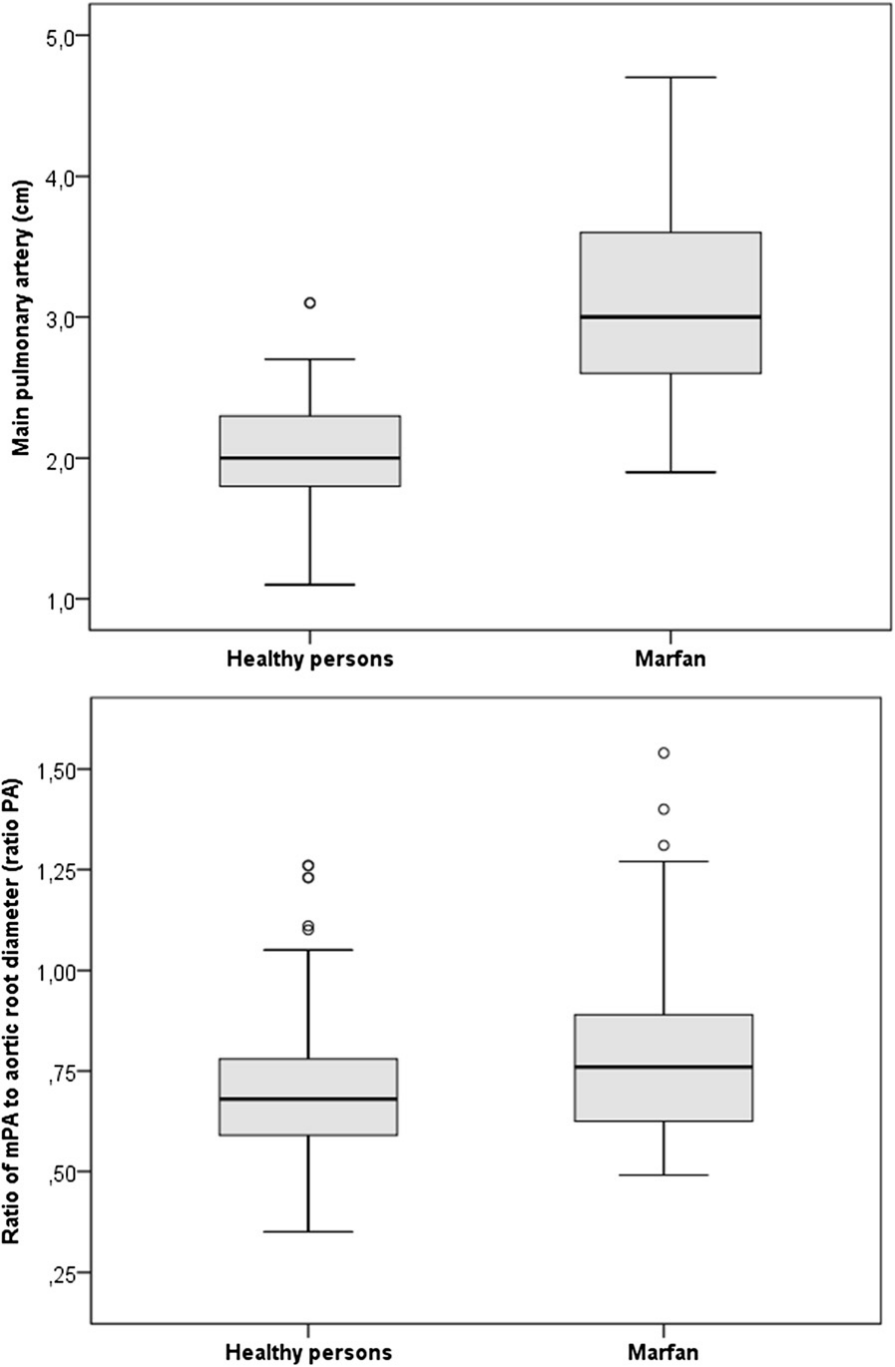
**The box-and-whiskers plots of main pulmonary artery diameters (MPA, upper panel) and of MPA ratios (lower panel).** Both, MPA diameters (*P* < .001) and MPA ratios (*P* < .001) were larger in Marfan syndrome than in normal adults.

With 2.1 ± .3 versus 3.1 ± .7 (*P* < .001), and with .71 ± .19 versus .8 ± .31 (*P* < .001), both MPA-d and MPA-r were smaller in normal persons than in MFS. MPA dilatation was present in 6 normal persons (4.9%) and in 68 MFS (69.4%; *P* < .001), MPA aneurysm was diagnosed only in MFS (15 patients; 15.3%; *P* < .001; Figure 3, upper panel), but no patient required surgery of MPA aneurysm. Using our threshold >1.05, MPA-r were increased in 6 normal persons (5.8%) and in 8 MFS patients (10.5%; *P* = .161; Table 3; Figure 3, lower panel).

Finally, linear regression analysis identified a relationship of increased MPA-d with increased aortic root diameters (regression coefficient (b) = .308; 95% CI .011–.604; *P* = .042), with reduced LVEF (b = −.021; 95% CI = −.036–.006; *P* = .006), with increased indexed LVEDD (b = .043; 95% CI = −.007–.079; *P* = .021), and with increased PASP (b = .023; 95% CI = .001–.045; *P* = .040). Conversely, no clinical manifestation of MFS, and no *FBN1* mutation characteristic related to MPA-d (Table 4).

## Discussion

Our study yielded five major results. First, we identified echocardiographic MPA-d >2.6 cm as upper normal limit of normal adults independent of age, sex, body height, body weight, BMI and BSA. Second, adults with MFS exhibited MPA dilatation in 69.4%, and MPA aneurysm in 15.3%, where no patient fulfilled criteria for surgical intervention. Third, MPA-r were increased in MFS as compared to normal persons, where a ratio >1.05 was identified as the upper normal limit. Fourth, MPA-d are similar in normal persons and MFS and may thus not be useful as diagnostic markers of MPA dilatation in MFS. Finally, MPA-d increased with PASP, aortic root diameters, reduced LVEF, dilated LVEDD but MPA-d did not relate to manifestations of MFS or to *FBN1* mutation characteristics.

In normal adults, upper limits of MPA-d were only defined on CT and MRI, where limits ranged between 2.8 cm [4], 2.96 cm [13], 3.01 cm [14], and 3.24 cm [15]. Our echocardiographic limit of 2.6 cm was slightly lower most likely because measurements on tomographic studies are usually obtained as outer diameters of a vessel rather than with the leading-edge method [25,26]. Our threshold may also be because some studies did not exclude PH [4,14,18]. Moreover, echocardiography measures MPA-d at other sites than on CT or MRI [2]. Most importantly, upper limits of MPA-d were defined differently as 90^th^ percentile [14], as 95^th^ percentile [13], or as diameters 0.5 mm above the maximum diameter [4]. One study used the maximum diameters measured in normal persons as upper normal limit of MPA-d [15]. Similarly, De Backer et al. identified 2.3 cm as cut-off of normal limit MPA-d on echocardiography, but they employed receiver operating characteristic curve analysis to separate normal persons from MFS rather than that they defined upper normal limits of MPA-d [2].

Using 2.6 cm as threshold we identified a high prevalence of MPA dilatation in MFS. One MRI study of 86 MFS patients listed different cut off values for MPA diameters [3], and used various methods to assess MPA dilatation: According to the old Ghent criteria for assessment of MPA dilatation [1], where a nomogram of the aorta made by Roman et al. [62] was employed to assess MPA dilatation, none of the patients were found to have dilatation of the MPA, and only seven patients had dilatation of the root of the pulmonary artery. Conversely, when using the cut off ≥3.0 cm as proposed by Bozlar et al. [13], the same authors found that 54% of MFS patients had MPA dilatation. Another study identified 76% of 50 MFS patients using 2.8 cm at the level of the bifurcation as threshold for MPA dilatation [4]. Interestingly, those two MRI studies with similar cut-off values as in our study identify MPA dilatation in 76% [4] and 54% of MFS [3] which was similar to our 69.4%.

We identify MPA aneurysms in 19% of MFS patients. The single study in the literature that also assesses the prevalence of MPA aneurysm in MFS reports an even higher frequency of 4 in 11 adults with MFS (36.4%) [73]. However, we did not identify any patient who required surgery for MPA-d >6.0 cm, or with complications such as regurgitation of the pulmonary valve, MPA dissection, or upper airway compression. In the series of Detrano et al. there was a single patient in 11 adults with MFS who exhibited an MPA-d >6.0 cm (9%) [73]. There is only casuistic evidence for complications of MPA aneurysm in MFS [49,50,52]. Interestingly, the Framingham Heart Study noted self-reported dyspnoea on exertion in 20% of normal persons with MPA-d above upper normal limits [14]. We did not assess symptoms in our MFS patients, but in summary there is little evidence that MPA aneurysm are a frequent source of complications in MFS.

The Framingham Heart Study defined their normal cohort of 706 persons similar to our criteria as individuals without obesity, arterial hypertension, current and past smokers, chronic obstructive pulmonary disease, history of pulmonary embolism, diabetics, cardiovascular disease, and heart valve surgery [14]. The study corroborated our finding that body height, body weight, BMI and BSA were not needed to define normal upper limits of MPA-d. We also did not identify significant sex-specific differences of MPA-d, whereas the Framingham study was the only study to define cut-off values for women and men. However, their normal population consisted of men aged >35, and women aged >40 years [14], whereas our normal population included much younger persons of both sexes. Moreover, studies with similar age distribution as in our study corroborated lack of significant differences between both sexes [13,19].

Our study defined >1.05 as upper normal limit of MPA-r on echocardiography, and thus corroborated thresholds form CT or MRI studies [16-19]. However, it is important to note that we measured aortic diameters at the level of the aortic sinuses, whereas MRI studies obtained aortic diameters as transverse axial diameters of the ascending aorta. Interestingly, with >0.9 the Framingham study reported an unusually low upper normal limit of MPA-r. But again, the Framingham study included only persons aged >35 years, and MPA-r is known to decrease with age, because only the proximal aorta dilates with older age [19]. Because of its age dependency, MPA-r is considered only of limited value for diagnosing PH [19].

We found MPA-r >1.05 with similar frequency in normal persons (5%) and in MFS (10.5%). This finding may be conceived as “pseudo-normal” MPA-r in MFS, where dilatation of MPA is neutralized by dilatation of the aortic root. Hence, MPA-r is not a good marker of MPA dilatation in MFS. However, we found a relationship of MPA-r with PASP in our MFS patients (regression coefficient (b) = .009; 95% CI .000–.017; *P* = .047), and hence, increased MPA-r may be useful to identify PH with increased risk for MPA aneurysm in MFS.

We identified a relationship of MPA dilatation with reduced LVEF, dilated LVEDD [74,75] and with increased PASP in MFS. Hence, MFS patients may develop high-pressure MPA aneurysm [52] when pulmonary hypertension develops from decreases of their myocardial function. Such decreases of myocardial function frequently develop in MFS from regurgitation of the aortic or mitral valve [76] or after repeated heart surgery [77,78].

Manifestations of MFS result from weakness of the tissue. Hence, it appears surprising that MPA-d did not relate to any other manifestations of MFS such as ectopia lentis, pneumothorax, dural ectasia, striae distensae, marked myopia, mitral valve prolapse, or Ghent scores of system involvement. A possible explanation is that additional factors are needed to manifest MPA dilatation. The association of enlarged MPA-d with increased PASP may indicate that PH is required as an additional factor to manifest formation of marked MPA aneurysm in MFS.

With *FBN1* mutations located in exons 24–32 MFS exhibits both, more pronounced aortic root disease [71] and more frequent ventricular arrhythmia events [7]. However, we did not identify a relationship of *FBN1* mutation characteristics with increased MPA-d. This finding supports the idea that MPA dilatation is driven by factors, which may not relate directly of the underlying disease mechanisms of MFS.

### Study limits

Some issues of our study may need discussion for appropriate estimation of our findings. First, our study provides normal echocardiographic values of MPA-d for adults, which can be used in any clinical setting independently of MFS. However, larger populations may be needed to corroborate our normative thresholds. Second, our MFS patients were sampled retrospectively with selection for availability of original echocardiographic recordings. Third, echocardiography may not be optimal for measuring MPA-d in adults, because diameters of the arterial walls are parallel to the direction of the scan plane and may thus not be measured precisely [27], or measurements may be inadequate as found in 5.3% of our MFS patients, and in 4% of MFS patients reported by De Backer et al. [2]. However, a recently published quality review of echocardiographic measurements found that intra-observer agreements for aortic root and MPA measurements were excellent [79]. Moreover, we did not include measurements of the pulmonary root because these were too variable both between the same observer and between different observers. Fourth, the levels and techniques of echocardiographic MPA measurements are not universally defined and hence our data may not be comparable with measurements in the literature. Fifth, we estimated PASP from non-invasive echocardiography without validation through right-heart catheterization [58]. Finally, our cross-sectional study was retrospective with reevaluation of routine echocardiographic examinations, where prospective longitudinal studies with standardized echocardiographic protocols may be needed to evolve the natural history of MPA dilatation in MFS.

## Conclusions

We establish 2.6 cm as upper normal value of echocardiographic MPA-d and 1.05 for MPA-r. In MFS, the prevalence of MPA dilatation is 69.4% and the prevalence of MPA aneurysm is 15.3%. However, patients may require MPA surgery only in scarce circumstances, most likely because formation of marked MPA aneurysm may require LV dysfunction and increased PASP.

## Abbreviations

BMI: Body mass index
BSA: Body surface area
cbEGF: Calcium binding calcium binding epidermal growth factor-like domain
COPD: Chronic obstructive pulmonary disease
CT: Computed tomography
*FBN1*: Fibrillin-1 gene
LVEF: Left ventricular ejection fraction
LVEDD: Left ventricular end-diastolic diameter
LVESD: Left ventricular end-systolic diameter
LTBP: Latent transforming-growth-factor beta–binding protein-like domain
MFS: Marfan syndrome
MPA: Main pulmonary artery
MPA-d: MPA diameter
MPA-r: MPA ratio
MRI: Magnetic resonance imaging
PASP: Pulmonary artery systolic pressure
PH: Pulmonary hypertension
Q-Q plot: Qnorm plot
